# Role of sucrose-dependent exopolysaccharides in the biofilm development of *Streptococcus mutans* revealed at the microscale level

**DOI:** 10.1128/aem.00009-26

**Published:** 2026-03-03

**Authors:** Ailin Huang, Xiaodan Li, Shangping Lu, Jingchao Zhang, Yujia Zheng, Miaoxiao Wang, Kun Zhao

**Affiliations:** 1 Institute of Fundamental and Frontier Sciences, University of Electronic Science and Technology of China 12599, Chengdu, Sichuan, China; 2 School of Life Science and Technology, University of Electronic Science and Technology of China 12599, Chengdu, Sichuan, China; 3 College of Ecology and Environment, Chengdu University of Technology 47908, Chengdu, Sichuan, China; 4 The Sichuan Provincial Key Laboratory for Human Disease Gene Study and The Institute of Laboratory Medicine, Sichuan Provincial People’s Hospital, University of Electronic Science and Technology of China 12599, Chengdu, Sichuan, China; Indiana University Bloomington, Bloomington, Indiana, USA

**Keywords:** dental caries, *Streptococcus mutans*, biofilm, exopolysaccharides, extracellular matrix, microscale

## Abstract

*Streptococcus mutans* (*S. mutans*) has a superior ability to rapidly metabolize sucrose into exopolysaccharides (EPS, mainly glucans), which serve as a critical virulence factor related to dental caries. Despite extensive research on sucrose-dependent EPS at the molecular and macroscale levels, however, the mechanisms underlying EPS effects at the microscale level remain poorly understood. Here, by employing bacteria tracking and fluorescence staining techniques, we investigated the role of sucrose-dependent EPS during biofilm development of *S. mutans* at the microscale level for both WT and Δ*gtfB* strains. The results showed that at the early stages of biofilm development, the sucrose-derived glucans enhanced the surface attachment of *S. mutans* through bamboo joint-like glucan patterns displayed on cell surfaces and altered their microcolony structures from loose 2D chains in Δ*gtfB* to dense-packed cell clusters in WT; then, after microcolonies formed, sucrose-dependent EPS promoted their development by speeding up the 2D-3D transition of WT microcolonies and affected final biofilm morphologies at the stage of biofilm maturation. Moreover, by tracking the long-time dynamic process of WT biofilm development at the microscale, the results demonstrated clearly the origin of liquid regions and their correlations with the structural and pH heterogeneity of biofilms. These findings establish sucrose-dependent EPS as dual-functional scaffolds—mechanically accelerating biofilm assembly, meanwhile, facilitating the formation of structural and pH heterogeneity inside biofilms that are critical for enamel demineralization, and thus provide insights for developing new anti-caries strategies.

**IMPORTANCE:**

*Streptococcus mutans* is a major pathogen in caries development due to its ability to rapidly metabolize sucrose into EPS. EPS serves as a major component of the *S. mutans* biofilm matrix, and previous studies mostly explored the effects of EPS on the macroscale. However, how EPS shapes *S. mutans* biofilm formation at the microscale is not well understood. By combining single-cell tracking with fluorescence staining techniques, we demonstrate that sucrose-dependent EPS governs the transition from 2D growth to 3D biofilm architecture and facilitates the formation of a liquid region at the bottom of the biofilm. These findings bridge a fundamental knowledge gap between the microscale organization and macroscale attributes of biofilms, offering novel perspectives for developing targeted anti-caries strategies.

## INTRODUCTION

Dental caries is currently one of the most widespread oral infectious diseases (1), and because of its high prevalence and substantial treatment costs, it has been identified by the World Health Organization as a major non-communicable chronic disease that requires prioritized prevention (2). The etiology of dental caries is closely related to dental plaque biofilms, where *Streptococcus mutans* (*S. mutans*) plays a pivotal role in cariogenesis (1–3).

*S. mutans* virulence is largely attributed to its ability to utilize sucrose. First, *S. mutans* has a strong ability to synthesize glucans from sucrose using glucosyltransferases (Gtfs). It can secrete three different Gtfs: GtfB, GtfC, and GtfD, encoded by *gtfB*, *gtfC*, and *gtfD*, respectively (4). In particular, GtfB synthesizes water-insoluble α-1,3-linked glucans (WIG), GtfC can produce both WIG and water-soluble glucans (WSG, mainly α-1,6-linked), and GtfD makes predominantly WSG. These secreted Gtfs, together with their synthesized glucans, promote the initial attachment of bacteria to the acquired enamel pellicle on the tooth surface (5, 6) and the building of the scaffold of dental biofilms (7–9). Second, *S. mutans* can ferment sucrose into acid while exhibiting high acid tolerance (7, 10, 11). Thus, these features together enable *S. mutans* to create an acidic, exopolysaccharide-rich (mainly glucans) microenvironment that promotes enamel demineralization (8). As a critical matrix component, sucrose-derived exopolysaccharides (EPS) determine biofilm pathogenicity by modifying physical and biochemical properties (12, 13). EPS can facilitate *S. mutans* colonization and accelerate microbial accumulation through promoting cell-to-cell and cell-to-surface adhesion (6, 8) and increasing the density of dental plaque biofilms, creating diffusion barriers that protect against antibiotics and host antimicrobial factors (14, 15) and restrict the diffusion of organic acids (16, 17). Given the importance of EPS, a considerable number of studies on the role of EPS in *S. mutans* biofilm formation have been reported (9, 18–22). Despite these advances, however, most of these investigations were performed from an aspect of either molecular level (e.g., gene regulations) or macroscale level (e.g., characterization of the whole cultures or colonies). Although recent methods such as expansion microscopy have begun to enable single-cell analysis of model biofilms including *S. mutans* (23), investigations that directly elucidate how EPS regulates *S. mutans* biofilm at the microscale remain limited. Thus, our understanding of the mechanisms underlying the EPS effects on *S. mutans* biofilms is still far from complete.

In this study, we aim to investigate the role of EPS during biofilm development of *S. mutans* at the microscale level by employing bacterial tracking techniques. We utilized *S. mutans* ATCC 25175, a model strain commonly used in caries research for its effective EPS production in a sucrose-enriched environment (24–26). We first investigated the effects of sucrose on bacterial surface attachment and microcolony formation for both WT and Δ*gtfB* strains. Using fluorescence staining techniques, we then revealed the role of sucrose-based glucans in the observed cell behaviors. Finally, through tracking the long-time dynamic process of biofilm development at the microscale, we revealed the evolution of heterogeneous structure and its associated pH distributions inside biofilms. The findings in this work will help to find new ways to battle dental caries.

## RESULTS

### Sucrose enhanced the surface attachment of *S. mutans* and altered their microcolony structures at the early stages of biofilm development

**Fig 1:**
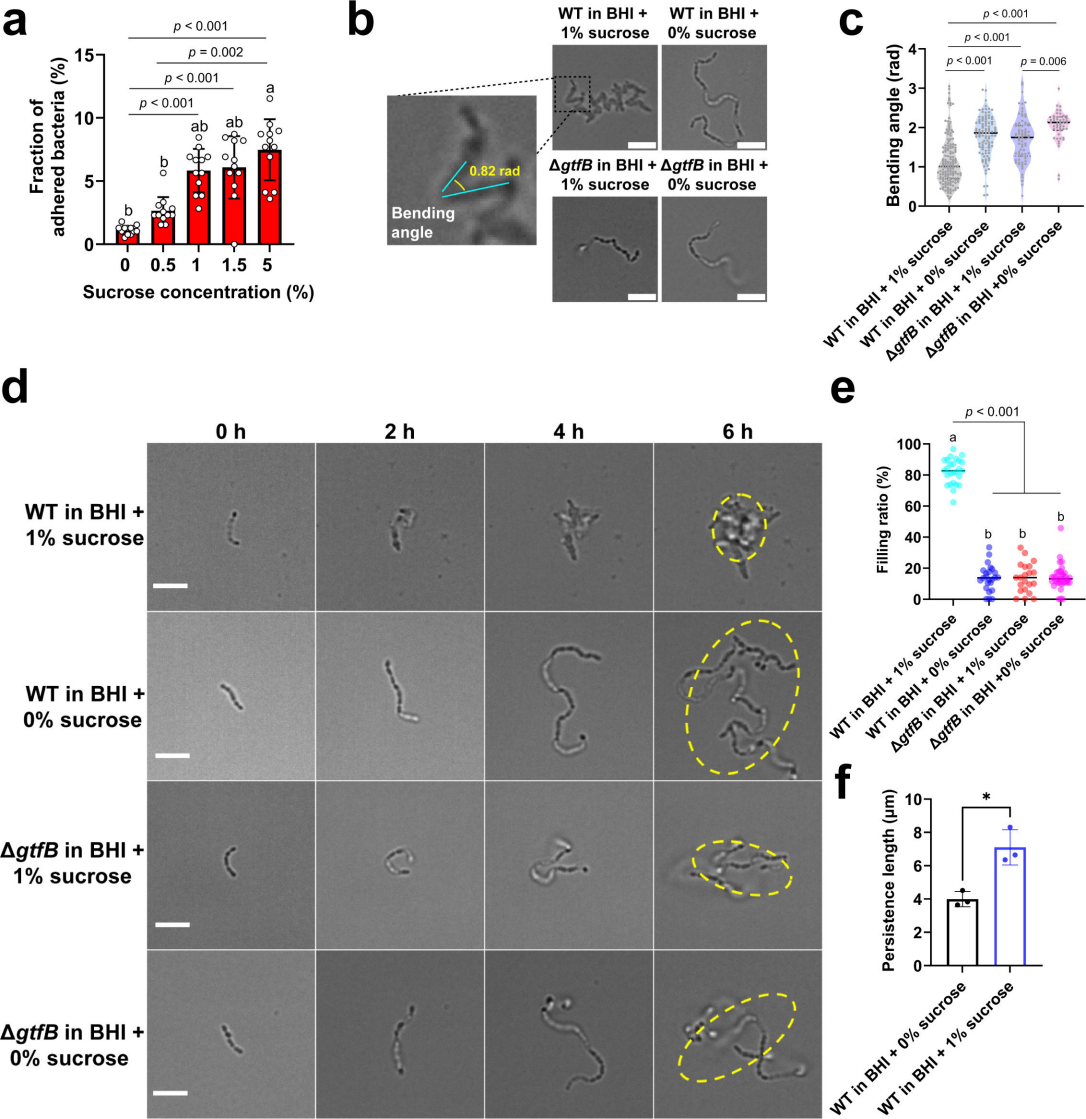
Effects of sucrose on bacterial behavior at early biofilm stages. (**a**) Fractions of surface-attached bacteria at varying sucrose concentrations. 12 fields of view from three independent experiments were analyzed (4 fields of view from each experiment). (**b**) Representative bright-field images showing the distinct morphological changes of attached WT and the Δ*gtfB* cell chains at 0 h and 3 h. The enlarged image (left) illustrates the definition of the bending angle, which is measured as the included angle formed by the two adjacent cells at their junction. Scale bar, 5 μm. (**c**) Quantification of the bending angle of attached cell chains. Lower angles indicate greater bending. The number of analyzed cell chains: *n* = 27 (6, 9, and 12 cell chains, respectively, from each experiment) for WT in BHI + 1% sucrose, *n* = 22 (6, 6, and 10 cell chains, respectively, from each experiment) for WT in BHI, *n* = 19 (8, 5, and 6 cell chains, respectively, from each experiment) for Δ*gtfB* in BHI + 1% sucrose, *n* = 16 (7, 4, and 5 cell chains, respectively, from each experiment) for Δ*gtfB* in BHI. (**d**) Different growth patterns of attached cells on a glass surface for WT and Δ*gtfB* in BHI + 1% sucrose and in BHI. The yellow ellipse represents the smallest enclosing ellipse of this microcolony. (**e**) The filling ratio of microcolonies was measured at 6 h after inoculation for WT and Δ*gtfB* with (BHI + 1% sucrose) and without (BHI) sucrose. The number of analyzed bacterial chains: *n* = 26 (7, 12, and 7 cell chains, respectively, from each experiment) for WT with sucrose, *n* = 24 (7, 7, and 10 cell chains, respectively, from each experiment) for WT without sucrose, and *n* = 21 (7 cell chains from each experiment) for Δ*gtfB* with sucrose, *n*= 34 (16, 9, and 9 cell chains, respectively, from each experiment) for Δ*gtfB* without sucrose. (**f**) Persistence length of free-floating bacterial cell chains after 15 h of cultivation with and without sucrose. The number of analyzed cell chains: *n* = 66 for WT in BHI + 1% sucrose and *n* = 64 for WT in BHI. Chains were chosen to have the same length of 3.00 ± 0.50 µm. Data are mean ± s.d. Statistical significance was measured using one-way ANOVA for (**a**), (**c**), and (**e**) and Student’s t-test for (**f**). Different lowercase letters (a, b, c) indicate statistically significant differences between groups (*P* < 0.05) .*P < 0.05. Scale bar, 5 μm.

To explore how EPS (mainly glucans) influences biofilm formation, we first examined the surface attachment of *S. mutans* wild-type (WT) cells at varied sucrose concentrations at the microscale level. The cell adhesion ratio (Fig. 1a) increased from 1.1% ± 0.4% (mean ± s.d.) in sucrose-free medium to 2.7% ± 1.1% at 0.5% sucrose and 5.9% ± 1.7% at 1% sucrose. Further increases in sucrose produced only minor, non-significant changes in the mean adhesion ratio (*p* > 0.999), suggesting that the effect of sucrose on the surface adhesion of cells approached saturation.

After attachment, *S. mutans* cells displayed distinct growth patterns with and without sucrose. In medium without sucrose, WT cell chains grew persistently along their body axis, and the divided daughter cells often continued their growth along the body axis of their mother cell, resulting in one-dimensional long cell chains. In contrast, for WT cells in medium containing 1% sucrose (BHI + 1% sucrose), after a cell division, the growth directions of one or both daughter cells often deviated largely from the body axis of their mother cell, thus leading to a two-dimensional flower-like cell cluster. In this study, we focused the analysis on insoluble glucans because the insoluble glucans primarily synthesized by *gtfB* using sucrose as a substrate are key for the 3D scaffold formation and structural integrity of *S. mutans* biofilms (4, 5, 27). Therefore, we constructed a *gtfB* mutant strain to verify this GtfB glucan-mediated mechanism (Fig. S1). By further testing the Δ*gtfB* mutant, we found that Δ*gtfB* cells in 1% sucrose displayed a similar growth pattern as WT cells in sucrose-free medium, suggesting that the observed sucrose-dependent growth behavior was through the synthesized glucans.

To quantitatively characterize this difference in chain morphology, we measured the bending angle between adjacent cells in the chains after 3 h of growth (before the transition to 3D growth). The bending angle is defined as the included angle formed by two adjacent cells at their junction (Fig. 1b). Under this definition, a larger angle indicates a more linear chain alignment, while a smaller angle corresponds to a sharper fold or buckling at the cell-cell junction. As shown in Fig. 1c, the mean bending angle for WT cells in BHI + 1% sucrose was 1.14 ± 0.62 rad (mean ± s.d.), which is significantly smaller than WT cells in BHI (1.78 ± 0.52 rad), Δ*gtfB* in BHI + 1% sucrose (1.70 ± 0.57 rad), and Δ*gtfB* in BHI (2.04 ± 0.39 rad) (*P* < 0.001). This result quantitatively confirmed that the presence of glucans promoted cell chains’ bending, leading to the observed dense flower-like clustering. However, no significant differences in bending angle were observed at 0.5%–5% sucrose concentrations (*P* > 0.05) (Fig. S2). To quantify cluster architecture, we measured the filling ratio (cell area divided by the minimal enclosing ellipse area) at 6 h (Fig. 1d), when microcolonies were distinct (a microcolony was defined as a cell chain/cluster with a cell number of ≥ 30 [28]). The filling ratio (Fig. 1e) measures how dense cells are packed inside a microcolony. The averaged filling ratio of a microcolony was 82% ± 8% (mean ± s.d.) for WT cells in BHI + 1% sucrose, which is much larger than 14% ± 8% for WT cells in BHI. It is also larger than the filling ratio for Δ*gtfB* cells in BHI + 1% sucrose and BHI, which is 14% ± 9% and 14% ± 8%, respectively. Given the adhesiveness of EPS, to further understand its effect on rigidity, we measured the persistence length of freely floating cell chains (see examples in Movies S1 and S2). Persistence length is a fundamental parameter in polymer physics; it represents the chain’s resistance to bending (longer persistence length indicates stiffer, more rigid chains). By measuring the persistence length of planktonic cell chains, we can assess how EPS production modulates the mechanical behavior of individual cell chains and contributes to the overall biofilm architecture. Figure 1f shows the results measured after 15 h of cultivation with and without sucrose. The persistence length of a freely floating cell chain cultured in BHI + 1% sucrose was 7.1 ± 1.1 µm, significantly longer than 4.0 ± 0.5 µm obtained in sucrose-free medium. In addition, EPS staining of WT planktonic chains showed that cells were encapsulated by EPS in the presence of sucrose, whereas no EPS encapsulation was observed in the absence of sucrose (Fig. S11). These results suggest that EPS stiffens planktonic cell chains, which agrees with our expectations given the adhesive nature of EPS, and the interaction of these rigid chains with the surface drives the observed bending and dense clustering.

### Sucrose markedly impacted the 2D-3D transition time of microcolony growth as well as the mature biofilm morphology

**Fig 2:**
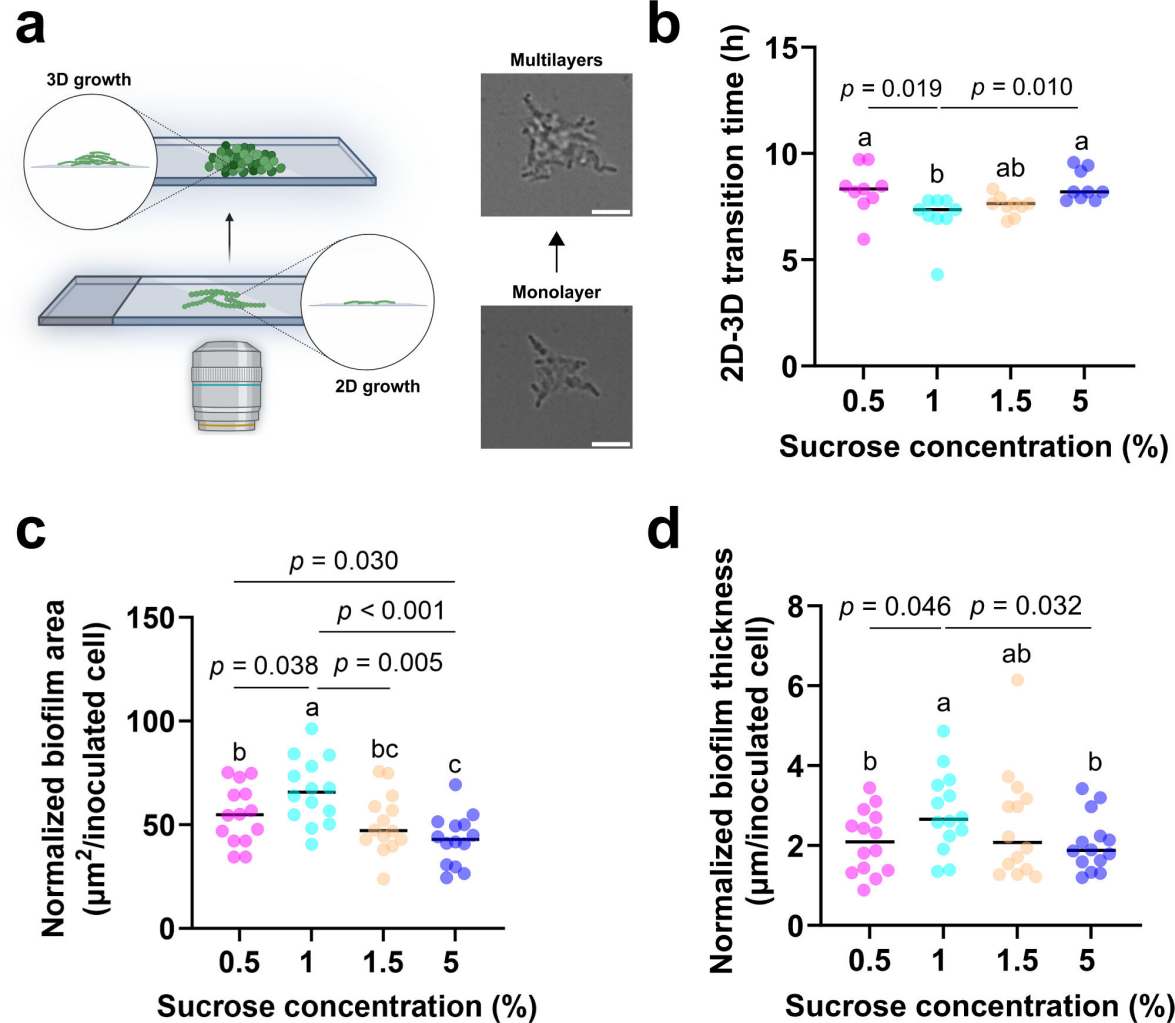
The sucrose concentration dependence of the 2D-3D transition time of microcolony growth and the matured biofilm morphology. (**a**) Schematic of 2D-3D transition of microcolony growth and representative bright-field images of monolayer and multilayer growth of microcolonies. (**b**) 2D-3D transition times of bacterial microcolonies at varying sucrose concentrations. Nine cell chains from three independent experiments were analyzed (three cell chains from each experiment). (**c**) Biofilm area and (**d**) biofilm thickness measured at different sucrose concentrations. The results were normalized by the total number of cells in chains that were initially inoculated. Fourteen cell chains from three independent experiments were analyzed (8, 3, and 3 cell chains, respectively, from each experiment). Statistical significances were measured using one-way ANOVA. Different lowercase letters (**a, b, c**) indicate statistically significant differences between groups (*P* < 0.05). Scale bar, 5 μm.

In the presence of sucrose, *S. mutans* microcolonies underwent a distinct transition from a monolayer (2D) to a multilayer (3D) structure. The 2D-3D transition time point is defined as the time point when the center region inside the microcolony with an area corresponding to the total area of 20 cells changed its appearance from black to white (corresponding to the thickness changes of the focal region) in bright-field images under our imaging conditions (Fig. 2a). We note that under our conditions, sucrose was required for the dense 3D structure formation. WT and Δ*gtfB* in 0% sucrose did not produce a detectable 2D-3D transition (Fig. S6), so no transition time is defined at 0% sucrose. Here, the region size of 20 cells was chosen arbitrarily, but we also tested 10 and 30 cells as the region size, and all results had the same trend (Fig. S3). The results (Fig. 2b) show that the transition time had a non-linear relation with the sucrose concentration and reached a minimum transition time of 7.0 ± 1.1 h at 1% sucrose.

As 3D microcolonies matured, dense biofilms would eventually form for the tested range of sucrose concentration from 0.5% to 5%. However, quantitative characterization after 12 h showed that the biofilms displayed different areas (Fig. 2c) and thicknesses (Fig. 2d) when different sucrose concentrations were used. Since *S. mutans* cells typically exist in a form of chains and to reduce variability due to the different chain sizes, we normalized area and thickness by the total initial cell count in chains. Similar to the results shown in Fig. 2b, the thickness and area of a biofilm also showed a non-linear relation with sucrose concentrations. Both the thickness and area of biofilms reached the maximum at 1% sucrose, with a value of 2.8 ± 1 µm/inoculated cell and 66 ± 15.7 µm^2^/inoculated cell, respectively. These results indicate that there is an optimal sucrose concentration for the formation of *S. mutans* biofilms, consistent with previous studies (18). Moreover, combining the results of Fig. 2b and d together seems to suggest that the 2D-3D transition time of microcolony growth was inversely correlated to the final matured biofilm properties (Fig. S4), as biofilms formed at 1% sucrose exhibited the shortest 2D-3D transition time, but had the largest thickness and area, whereas those at 5% sucrose exhibited the longest transition time and had the smallest thickness and area. Growth curves (Fig. S5) showed similar growth for 1%–5% sucrose (minor slowing at 0.5%), indicating that the effect of sucrose on cell growth played a minor role in the observed different behavior in the transition time and matured biofilm properties at different sucrose concentrations.

### Dynamic measurements of glucan production revealed its correlation with the phenotypic observations of *S. mutans* during biofilm development

**Fig 3:**
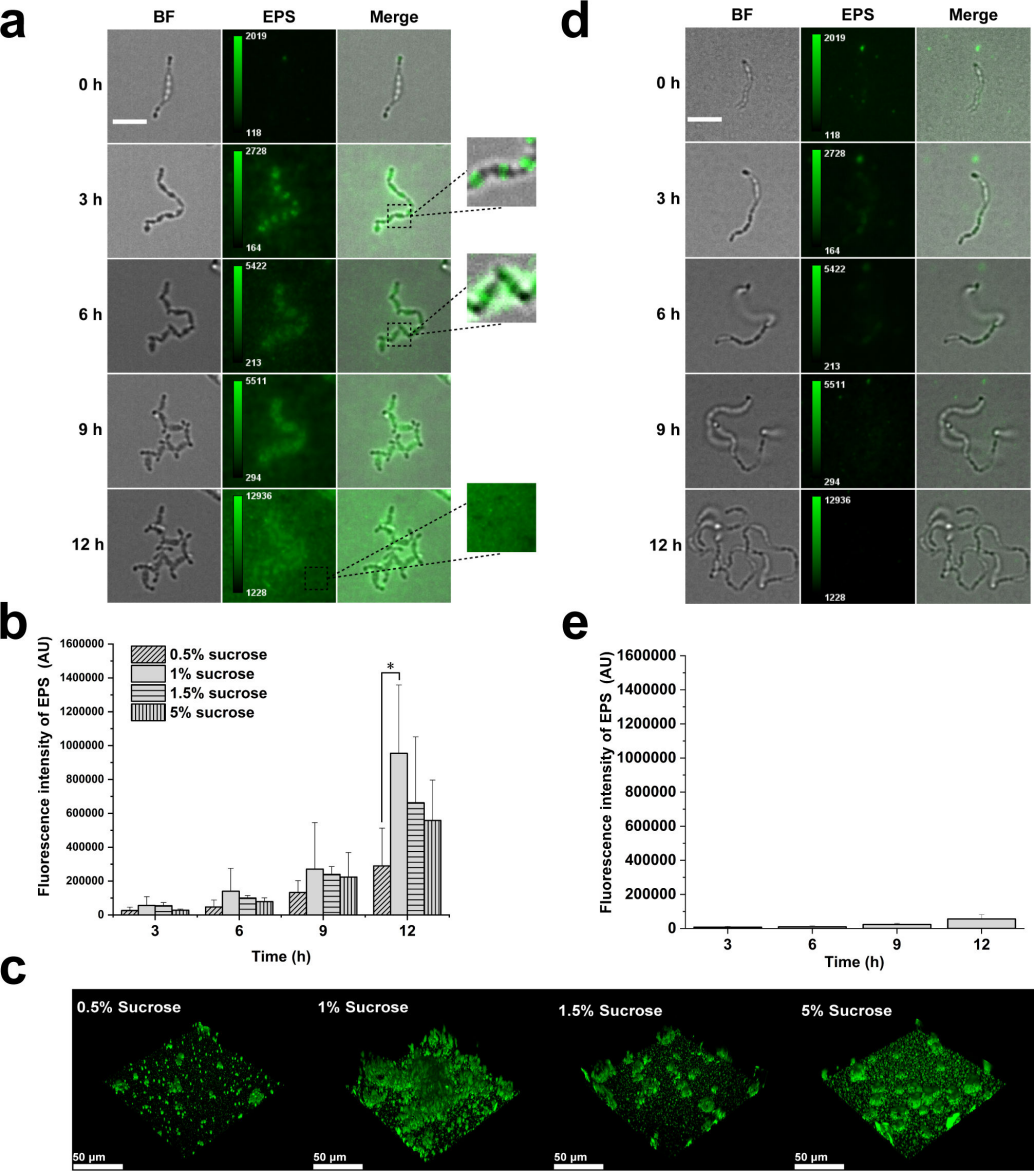
Dynamic measurements of glucan production. (**a**) Time-lapse micrographs illustrating the glucan production of *S. mutans* WT cells in BHI + 1% sucrose. The enlarged insets at 3 h and 6 h were manually adjusted for enhanced presentation. Color bars represent the fluorescence intensity range. Scale bar, 5 μm. (**b**) The total fluorescence intensity of glucans produced by WT cells in BHI + 1% sucrose in the observed fields was measured at different time points at various sucrose concentrations. (**c**) Representative 3D-rendered images of *S. mutans* WT cells in BHI + 1% sucrose biofilms grown for 20 h at different sucrose concentrations. (**d**) Time-lapse micrographs illustrating the glucan production of *S. mutans* Δ*gtfB* cells in BHI + 1% sucrose. Color bars represent the fluorescence intensity range. To facilitate direct visual comparison, the range of gray values of the fluorescence images at each time point was manually adjusted to be consistent with (**a**). Scale bar, 5 μm. (**e**) The total fluorescence intensity of glucans in the observed fields was measured at different time points of Δ*gtfB* cells in BHI + 1% sucrose. Data are shown as mean ± s.d. (three fields of view from three independent experiments were analyzed). Statistical significances were measured using one-way ANOVA. *, *P* < 0.05.

It is well known that *S. mutans* can use sucrose to synthesize glucans, which are important exopolysaccharides in dental biofilms (29). To elucidate the mechanism underlying these phenotypes, we tested the biofilm development of WT cells in BHI and Δ*gtfB* cells in BHI + 1% sucrose. The results (Fig. S6a) show that they both formed a loose thin biofilm, in contrast to the typical dense 3D structure formed by WT in BHI + 1% sucrose. These results further support the key role of glucans in these behaviors. Next, we utilized fluorescently labeled dextran conjugates as a primer and acceptor for Gtfs (particularly GtfB) to label synthesized glucans *in situ* (19) in a flow-cell chamber. All imaging acquisitions were performed under identical settings to ensure quantitative comparability. Figure 3a shows an example displaying glucan distribution along a cell chain during the whole attachment process of the chain. At the initial stage (0 h in Fig. 3a), glucan signals co-localized with attached cells, supporting the role of EPS in surface adhesion. Then, these early-attached cells may function as “anchor” cells, which would prevent unattached cells in the same chain from moving away and thus facilitate their attachment to the surface (Movie S3). Over time (3 h), discrete fluorescence patches appeared at the cell septum, creating a “bamboo joint-like” pattern. Given the adhesive properties of glucans, these discrete bamboo joint-like patches would help to anchor the entire bacterial chain firmly to the surface. As cells continued to grow and divide, at 6 h, fluorescence regions expanded around cells, and the total fluorescence intensity also increased, suggesting a continuous production and accumulation of glucans. Correspondingly, the anchored chains began to buckle at cell junctions (see one example shown in the enlarged window at 3 h and 6 h in Fig. 3a), consistent with the bending analysis in Fig. 1c. Dynamics of glucan production (Fig. 3b) paralleled biofilm development, with a sharp increase in fluorescence intensity between 9 and 12 h, corresponding to significant accumulation in cell-free regions. This suggests active glucan synthesis by extracellular Gtfs (30). We note that the microcolony formation in Fig. 3a was significantly delayed when compared with Fig. 1b, which is very likely due to the damage to cells caused by lasers during fluorescence imaging.

The dynamics of glucan production were measured at different sucrose concentrations. As shown in Fig. 3b, all the results showed a similar trend, that is, the fluorescence intensity, which corresponds to the amount of glucans produced, increased with time. Particularly, there was a sharp increase in the fluorescence intensity from 9 to 12 h, consistent with the observation that there were much stronger fluorescence signals in the cell-free regions at 12 h than at 9 h, as displayed in Fig. 3a. Quantitatively, the amount of glucans produced during the same time period followed a non-linear relation with the sucrose concentration, with a maximum at 1% sucrose. A similar trend in the sucrose concentration dependence trend was observed for a more developed biofilm at 20 h, as shown in Fig. 3c, where the biofilm formed at 1% sucrose had the largest total fluorescence intensity. These results are consistent with the results shown in Fig. 2.

As *S. mutans* can synthesize both WIG and WSG, to further reveal the role of WIG in the biofilm development, we monitored the EPS production of the Δ*gtfB* mutant in BHI + 1% sucrose at both micro- and macro-levels. The microscopic tracking results (Fig. 3d) show that the loss of *gtfB* significantly impaired the bacteria’s ability to produce sufficient glucans, which are essential for firmly adhering to surfaces. The typical bamboo-like distribution of EPS was no longer observed. Consequently, the fluorescence intensity (Fig. 3e) was nearly 10 times lower compared to the WT cell in BHI + 1% sucrose (Fig. 3b). In the absence of sucrose, neither WT nor Δ*gtfB* cells exhibited the bamboo-like EPS distribution, and both produced negligible amounts of EPS (Fig. S7). The macroscopic results (Fig. S6b) show that Δ*gtfB* has a more uniform distribution but with less intensity compared with WT under the same medium condition, in agreement with the biofilm appearance of the two strains demonstrated in bright-field images (Fig. S6a). These phenotypic differences between WT and Δ*gtfB* are consistent with previous reports (9). As a negative control, when there was no sucrose presence, sucrose-based glucan production was abolished, and essentially no fluorescence-labeled glucans were observed (Fig. S6). These results support that WIGs synthesized by GtfB play an important role in the observed biofilm development process of *S. mutans*.

### Biofilm tracking at the microscale revealed the evolution of heterogeneous structure and its associated pH distributions inside biofilms

**Fig 4:**
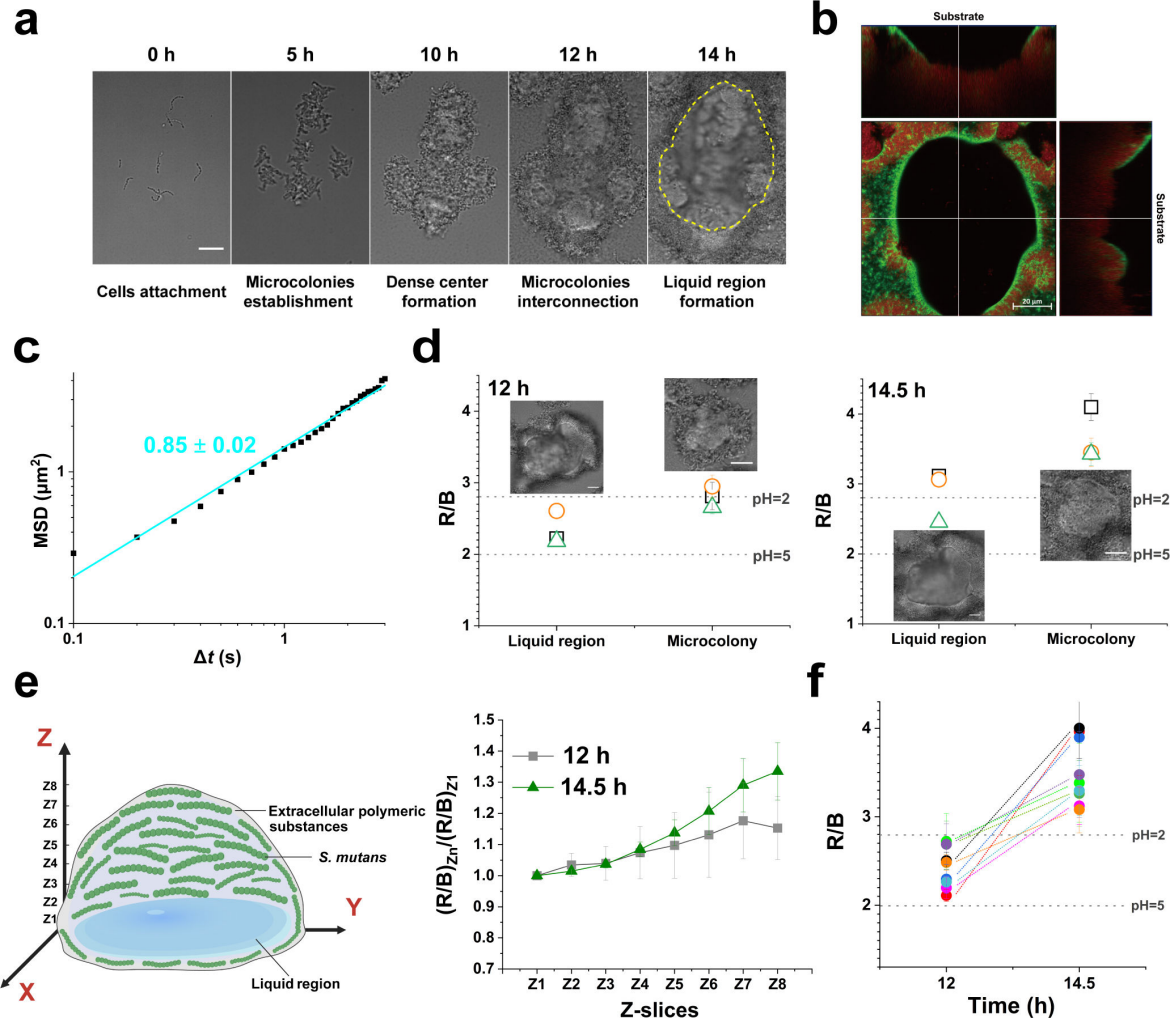
The evolution of heterogeneous structure and its associated pH distributions inside biofilms. (**a**) Tracking of a biofilm development process, revealing the liquid regions formed inside the biofilm. (**b**) A typical section image of a 14.5-h-old biofilm with both EPS (green) and cell membrane (red) stained. Horizontal sectioned image of the biofilm (square) close to the surface, and two vertical sectioned images (rectangle) of the biofilm are shown. (**c**) An example of a mean squared displacement (MSD) curve measured for planktonic *S. mutans* WT cells in liquid regions. The solid line is the fitted curve of MSD data in the log-log plot. (**d**) Measured fluorescence intensity ratios of red and blue channels for a liquid region and its surrounding microcolonies. Data points with each different symbol represent a repeat. An exemplar image for each region was also shown. The corresponding R/B values of pH = 2 and pH = 5 obtained from a calibration curve (Fig. S10) were indicated. Note that the working range of the pH probe is stated to be 2–14. (**e**) A schematic (left) showing a typical colony region that encloses a liquid region. From bottom to top, the colony region is divided into eight slices from Z1 to Z8, and the pH measurements along the biofilm height were performed at selected regions of each slice (right). Data are shown as mean ± s.d. (nine biofilms from three independent experiments were analyzed, three cell chains from each experiment). (**f**) pH of a colony containing liquid regions was measured at two time points. Each data point was the averaged value of Z1–Z8 slices of each colony measurement. Each identical color represents the measurements at two time points for the same colony, and a total of nine repeats were shown. Scale bar, 10 μm. Dotted lines are guides to the eye.

Biofilms with typical 3D structures are heterogeneous (8, 31, 32). However, the dynamics of the heterogeneous structures at the microscale are poorly understood. Taking advantage of the bacterial tracking techniques, we monitored the structural dynamics during the whole biofilm development of *S. mutans* at the obtained optimal sucrose condition for biofilm formation (BHI + 1% sucrose). Figure 4a shows a typical process of mature *S. mutans* biofilm development covering the period from initial inoculation to the mature biofilm. The process followed the common picture of biofilm development revealed in other bacteria, for example, *P. aeruginosa* (33, 34), that is, initial attachment (0 h), followed by microcolony formation (5–6 h), and more developed 3D colonies at later time points. While these stages are well established, we captured novel subsequent events critical for structural heterogeneity. As microcolonies of *S. mutans* continued to grow and expand, neighboring microcolonies would eventually come to contact (35). At this point, we observed that the strong EPS-mediated cohesion within microcolonies and their adhesion to the substrate led to a distinct instability. A further expansion of neighboring colonies would induce buckling to form bulges at places where neighboring colonies met, similar to the phenomenon in geology where two plates are extruded to form mountains. To validate that this structural reorganization was driven by mechanical buckling, we tracked the movement of fluorescent beads in the biofilm. As predicted by the buckling hypothesis, beads on the surface were incorporated into the biofilm by EPS adhesion and remained attached on the surface until colliding‌ and merging with neighboring microcolonies. The beads adhered to the biofilm are subsequently carried upward with the developing bulges (Fig. S8a). Quantitative tracking of the fluorescent beads’ vertical positions confirmed this time-dependent formation and upward movement (Fig. S8b; Movies S4 and S5), providing direct evidence for mechanical stress-induced deformation during biofilm development. As the two contacting neighboring colonies were lifted upward gradually to form bulges, the space between colonies and the substrate formed liquid regions, which were enclosed by merging colonies (see the image at 14 h in Fig. 4a; Movie S4).

There are several lines of evidence to support that the enclosed region is liquid-like. First, our confocal scanning images with biofilm EPS stained with Alexa Fluor 488–dextran conjugate and bacterial cells labeled with FM4-64 revealed a clear void at the bottom of the biofilm that contains neither EPS nor bacterial cells (Fig. 4b; Movie S6). Second, in these regions, we observed planktonic cells that were jiggling in a diffusive type of motion (Movie S7). To quantitatively characterize the cell motion in these regions, we calculated the mean squared displacements (MSDs), which measure to what extent a cell motion deviates from a typical random diffusive motion of cells from their trajectories by bacteria tracking techniques, as previously described (36). From the MSD curve (Fig. 4c), we can see that MSD increased with time, supporting that cells were in a liquid-like environment. Moreover, by fitting the MSD curve in a log-log plot, a slope of 0.85 ± 0.02 could be obtained, which is smaller than 1 (the slope of an MSD in a log-log plot for a Brownian random diffusion), suggesting that bacterial cells in these liquid regions performed a sub-diffusive-like motion.

The EPS matrix acts as a diffusion barrier that limits penetration of charged ions (8, 30, 37). However, uncharged solutes such as sucrose may readily diffuse into the biofilm and be utilized for acid production by the embedded bacteria (38, 39). The consequence of this barrier function would lead to a heterogeneous distribution of pH in biofilms. Particularly, given the existence of liquid regions that were enclosed by colony regions, we hypothesized that the pH of liquid regions would be different from that of their surrounding colony regions. To test this hypothesis, we measured *in situ* pH by using a ratiometric dye (8, 27, 37). We first measured and compared the pH of liquid regions with their surrounding microcolonies. The pH value was measured using a pH probe based on the ratio of fluorescence intensity of red and blue channels, with a working pH range of 2–14. The actual measured ratios can fall out of the working range, resulting in non-determinable pH values. To be consistent, we presented the ratios of red to blue fluorescence intensities (R/B) instead of converted pH values. The results (Fig. 4d) show that the R/B ratio was lower (i.e., pH was higher) in liquid regions compared to their surrounding colony regions. For a longer incubation time, R/B ratios in both regions were increased, but the trend that the liquid region had a lower R/B ratio than the surrounding colony region remained. These results support our hypothesis.

Considering the diffusion barrier inside microcolonies, it is conceivable that pH within a microcolony may also be non-uniformly distributed. Therefore, we further measured the pH distribution along the vertical direction from the bottom of an enclosed liquid region all the way up to the top of the surrounding colony region. Figure 4e shows the measured results at eight selected slices Z1–Z8 that were evenly distributed along the vertical direction and were normalized by that of Z1. In the specific case shown in Fig. 4e, Z1, Z2, and Z3 values were measured in the liquid region, while the rest of the slices were in the colony region (i.e., cell-matrix region). From Fig. 4e, we can see that there was not much change from Z1 to Z3, as they were all in a liquid region (thus should be uniform due to the relatively free diffusion of ions). However, from Z4 to Z8, the normalized R/B ratio clearly showed an increased trend, suggesting that the colony became more acidic from bottom to top. This trend is stronger at a longer incubation time, as demonstrated between the results at 12 h and at 14.5 h. The increase in acidity with incubation time was also confirmed by comparing the averaged values of all slices measured at 12 h and at 14.5 h for the same sampling region of the biofilm (Fig. 4f). We note that the liquid regions that we studied here were fully enclosed regions, that is, they were spatially isolated from the external liquid medium by surrounding colony regions, which was also supported by the statistically different R/B ratios between the liquid region (Z1–Z3) and the external liquid medium region measured at 12 h (Fig. S9).

## DISCUSSION

**Fig 5:**
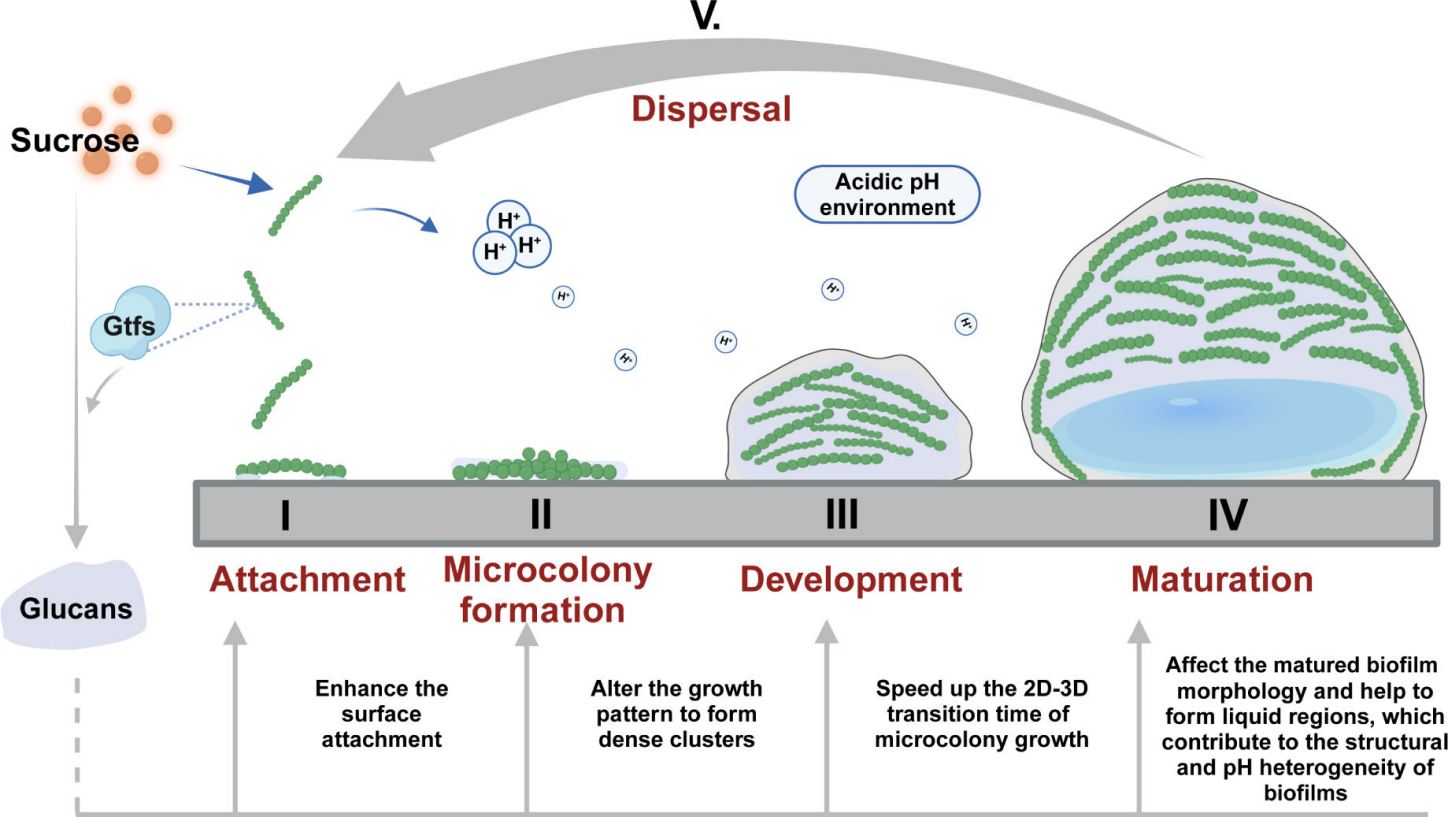
Schematic showing the effects of sucrose-dependent EPS in the biofilm development of *S. mutans*.

In this study, by employing bacterial tracking techniques and fluorescence staining, we have investigated how sucrose-dependent EPS (mainly glucans) shapes *S. mutans* biofilm development at the microscale. While prior studies have described many of the endpoint phenotypes associated with EPS production, our study revealed that the EPS is not merely a structural component but a dynamic regulator that dictates the developmental trajectory of *S. mutans* biofilms, and this regulatory action cannot be directly accessible to endpoint assays. Specifically, we showed that sucrose-dependent EPS (i) enhanced cell chains adhesion, (ii) altered the mechanical properties of planktonic cell chains (increased chain rigidity), (iii) promoted bending and breaking of substrate-anchored cell chains, and (iv) sped up the 2D-3D transition time of microcolony growth and ultimately produced the spatial configuration of structural and chemical heterogeneities (Fig. 5).

At the stage of surface attachment, fluorescence labeling of newly produced glucans shows progressive, discrete “bamboo-joint” coating of cell surfaces (Fig. 3a), which correlates with an increased fraction of stably attached cells (Fig. 1a). Our single-cell chain tracking demonstrates that the cells that attached earlier would help other cells in the chain to finally become surface-attached, consistent with a local adhesive reinforcement mechanism. The adhesion forces between *S. mutans* WT cells and the substrate in 1% sucrose have been reported to be in the order of hundreds of piconewtons (40, 41). By contrast, the adhesion force is reduced by 4.5- to 7-fold in the absence of sucrose or Δ*gtfB* mutants was used (40), which clearly demonstrated the important role of glucans in cell-substrate adhesion.

At the micromechanical scale, glucan-mediated anchoring of cells produces distinct growth patterns of attached chains. As cells divide, the glucan-mediated fixation to the substrate converts axial growth into mechanical stress, inducing buckling and bending at cell-cell junctions. This growth-induced deformation, a phenomenon previously reported in *Bacillus subtilis* (42), is a key microscale mechanism that breaks linear chains and initiates the formation of densely packed, multi-layered clusters, building the foundation for 3D development. In contrast, for the case of no glucans, cells would relatively easily extend along their body axis as cells loosely settled on the surface. To quantify this mechanical effect, our calculations revealed that in the presence of glucans, chains of identical geometric length showed higher persistence length (greater rigidity) (Fig. 1d), indicating that EPS can stiffen the planktonic cell chains (Fig. S11) and alter how growth stresses are accommodated.

A critical quantitative finding of our work is the demonstration that EPS actively accelerates the 2D-3D transition of microcolony. The surface-formed glucans by GtfB contain greater amounts of 3-linked glucose and more branch points, primarily consisting of 3,6-linked glucose and 2,3-linked glucose than glucans formed in solutions (43). This would make them highly insoluble and structurally rigid, which could promote the vertical growth of microcolonies (9). We propose that the abundance of insoluble glucans not only provides a scaffold but also generates internal mechanical stresses that actively push the biofilm upward, a mechanism distinct from motility-driven transitions in other species (44). The most direct evidence for this mechanical role is the formation of “liquid regions.” Our bead-tracking experiments confirmed that these voids result from a buckling instability. The strong adhesion of the EPS combined with the outward pressure from growing microcolonies induces buckling to form bulges at places where neighboring colonies meet and the formation of enclosed liquid regions (Fig. S8; Movies S4 and S5). These results provide a dynamic, mechanistic origin for the hollow structures previously observed in SEM observations of *S. mutans* biofilms (32, 41), and strengthen the mechanistic link between EPS-mediated adhesion, growth stress, and microscale structural reorganization.

The spatial organization of EPS also governed the chemical microenvironment, leading to pronounced pH heterogeneity (21, 22, 27, 37, 45, 46). We measured pronounced pH heterogeneity, and enclosed liquid regions at the biofilm bottom are consistently less acidic than the surrounding cell-matrix regions, while the cell matrix shows increasing acidity from bottom to top. We attribute this vertical gradient to three factors under our flow-cell conditions: (i) continuous nutrient flow sustains higher metabolic activity at the microcolony top (near the medium interface) (47), (ii) enclosed liquid regions at the bottom act as dilution reservoirs that buffer proton accumulation, and (iii) EPS-mediated differences in matrix porosity/hydration modify local proton diffusion. These findings appear to contrast with several previous reports conducted in static buffer systems, where restricted diffusion could trap protons near the substrate and produced an inverse gradient (8, 37, 46). We believe that this discrepancy is likely due to different experimental conditions employed. In our flow-cell system, cells at the upper interface are metabolically more active due to continuous nutrient supply, showing increasing acidity from bottom to top. Our results (Fig. 4f) are consistent with reports that pH levels can drop from neutral pH 7.0 to acidic values below pH 3.0 following continuous sugar exposure (48). Both the cell-matrix region and the liquid region had a pH value well below the critical pH of 5–5.5, at which the demineralization of the tooth begins (2, 49). However, their effects on tooth decay may be different. The cell-matrix region may trap ions to facilitate remineralization (2). While the liquid region allows unconstrained ion diffusion, potentially diluting dissolved minerals and hindering remineralization.

Beyond heterogeneity, the liquid region may also serve as a flow channel for the efficient exchange of substances within the biofilm. While we focused on enclosed regions relevant to acid accumulation, open liquid regions connected to the external medium could efficiently transport nutrients and metabolites of cells between cells embedded inside biofilms and the external fluid medium, like other species (50, 51). Additionally, recent work suggests that the EPS matrix can generate osmotic pressure that induces water uptake at the biofilm bottom, driving localized swelling and the translocation of bacterial micro-aggregates to new surface positions (24). These mechanisms reveal the complex functional roles of EPS-mediated liquid regions and should be considered in future work addressing EPS-targeted control strategies.

Our experiments used a monospecies *S. mutans* model, which cannot capture interspecies EPS consumption, matrix remodeling, or competition that occur in dental plaque. In multispecies biofilms, EPS produced by *S. mutans* can be metabolized or degraded by neighboring species, function as a shared scaffold that benefits multiple taxa, or be competitively displaced by alternative matrix components. Each of these interactions could substantially modify the 2D-3D transition of the microcolonies and the persistence of enclosed liquid regions. Therefore, further studies may be required to assess how the EPS-mediated biofilm formation is modulated by interspecies interactions.

In conclusion, by employing bacteria tracking and fluorescence staining techniques, we have revealed the roles of sucrose-dependent EPS (mainly glucans) at the different stages of biofilm development of *S. mutans* at the microscale level. Our results indicate that EPS regulates biofilm development by enhancing surface attachment of cells, forming dense clusters, speeding up the 2D-3D transitions of microcolony, and affecting the final morphology of mature biofilms. In addition, by monitoring the biofilm development at the microscale, we demonstrated clearly the origin of liquid regions and their correlations with the structural and pH heterogeneity of biofilms.

We believe that these micro-scale dynamic insights and the proposed follow-up mixed-species tests will help bridge mechanistic understanding from simplified models to the complex ecology of dental plaque. This will provide valuable insights to develop EPS-based new methods for the control of dental caries.

## MATERIALS AND METHODS

### Bacterial strains and growth conditions

*S. mutans* ATCC 25175 and *Escherichia coli* (*E. coli*) DH5α were used in this study. *S. mutans* WT and Δ*gtfB* were cultivated in brain heart infusion (BHI) broth and grown statically at 37°C under aerobic conditions. *E. coli* was grown in Luria-Bertani (LB) broth and in a shaking incubator at 200 rpm or on LB agar plates in an incubator at 37°C. Antibiotics were used at the following concentrations: 1 mg/mL spectinomycin for *S. mutans* and 100 μg/mL spectinomycin for *E. coli*. Cell density of *S. mutans* and *E. coli* was measured at optical density at 600 nm (OD_600_) using a spectrophotometer (AOE, Shanghai, China), respectively. *S. mutans* growth curves were detected by measuring the optical density of cultures at 600 nm. Overnight cultures of *S. mutans* were diluted in BHI with different sucrose concentrations, preheated to 37℃, and monitored every hour.

### Mutant construction

The *E. coli* DH5α strain was used for plasmid construction and amplification. All the primers were synthesized by Sangon Biotech. (Shanghai, China). Primers used in this study are listed in Table S1. Δ*gtfB* was derived from *S. mutans* using PCR ligation mutagenesis with the insertion of the *aad9* gene to replace the targeting genes. Briefly, the upstream and downstream fragments of *gtfB* were amplified from the *S. mutans* genome using PCR and then ligated into the pFW5 plasmid (52). The resulting plasmid was digested with SpeI and transformed into *S. mutans* for homologous recombination. The constructs were validated by colony PCR and confirmed by Sanger sequencing.

### Transformation of *S. mutans*

Transformation was done as previously described (53) with modifications. An overnight culture of *S. mutans* grown in BHI was diluted 1:40 in BHI supplemented with 5% heat-inactivated horse serum (Gibco, USA). Cultures were incubated at 37 °C to reach OD_600_ ~ 0.2, and then competence-stimulating peptide (CSP, amino acid sequence: NH2-SGSLSTFFRLFNRSFTQALGK-COOH, purity > 95%) was added to a final concentration of 1 μg/mL to induce genetic competence for transformation, together with 4 μg of DNA. 200 μL of the suspension was spread onto BHI agar plates containing 1 mg/mL spectinomycin and incubated anaerobically for 24 h.

### Flow cell assembly and biofilm formation

Flow cells (Ibidi Sticky-Slide VI 0.4) were purchased from the ibidi company. A syringe fitted with a 0.22 μm filter (Millipore) is connected to the inlet of the flow cell using silicone tubing. The entire system was then sterilized overnight with 3% H_2_O_2_ at a flow rate of 3 mL/h. After sterilization, the system was washed overnight with autoclaved, deionized water. Before inoculating the bacteria into the flow cell, the system was flushed for 5 min at a flow rate of 30 mL/h with culture medium using a syringe pump. Subsequently, the medium flow was stopped, and 300 μL of a diluted bacterial culture (OD_600_ ~ 0.1) was injected directly into the flow cell channel using a 1 mL syringe with a needle. A 20-min incubation period was allowed for the cells to attach to the surface, followed by a high flow rate of 24 mL/h for 20 min to wash out any floating cells. The flow rate was then set to 1.2 mL/h, and image recording was started. The flow cell experiments were conducted at 37°C.

For the tracking of fluorescent beads, *S. mutans* (OD_600_ ~ 0.1) was mixed with 5 µL of fluorescent beads (2.5% wt/vol, 3 µm, excitation 540 nm, emission 580 nm) into fresh BHI. The resulting mixture was injected into a flow cell and cultured at 37°C for 15 h in BHI + 1% sucrose. Bright-field images were acquired every 5 min, Z scans were performed once per hour, and the Z position (height above the surface) of each bead was recorded.

### Fluorescent staining

EPS labeling was achieved using Alexa Fluor 488-labeled dextran conjugate (molecular weight, 10 kDa; absorbance/fluorescence emission maxima of 495/519 nm; Molecular Probes, Invitrogen Corp., Carlsbad, CA). A final concentration of 1 μM dye was added to the culture medium from the beginning and continuously injected into the flow cell chambers using a syringe pump for 12 h. As previously described (8), bacterial cells were not stained at this concentration. Cell membrane staining was obtained by injecting FM4-64 (10 μM final concentration, Molecular Probes) into the flow cell chamber for 10 min in the dark (without flow). The dye-containing culture medium and sterile PBS solution were placed in separate syringes, each connected to its own syringe pump. A Y-connector was used to link the dye and PBS solution. Before fluorescence imaging, the dye injection was stopped, and PBS was injected at a flow rate of 24 mL/h for 5 min to remove excess dye.

### Ratiometric pH measurements and visualization

*In situ* pH in the biofilms was monitored by confocal microscopy with the ratiometric dye AIE-pH-R01 Probe (AIE Institute). AIE-pH-R01 is an aggregation-induced emission (AIE) effect (54) based on a pH probe that responds to pH and displays acidic and basic intervals in red and blue fluorescence. The excitation wavelengths of the pH probe are 405 nm and 488 nm, and the fluorescence intensity of both emission wavelengths within 417–477 nm (blue) and 575–625 nm (red) intervals, and the ratio of fluorescent intensity (red/blue) within each biofilm image was measured using Image J and its calculation tools. The confocal images were captured using a Nikon Ti2E + Xlight V3 microscope (Nikon Corporation, Tokyo, Japan) equipped with a 100× oil objective. The image size is 143.36 μm × 143.36 μm (2,048 × 2,048 pixels). For confocal microscopic calibration, PBS solutions (adjusted to pH values ranging from 2 to 7 in 1 pH unit increments), containing AIE-pH-R01 Probe at a concentration of 10 μM, were imaged in flow cell chambers. The calibration of the AIE-pH-R01 Probe is shown in Fig. S10. After 12 h of biofilm growth, the pH of the culture medium in the flow cell chamber was measured to be 4.0. Then, we followed the product instructions and prepared a working solution of the probe at a final concentration of 10 µM using PBS at pH 4.0 and injected 200 µL of this solution into the flow cell chamber. This would replace the original medium in the chamber while keeping the pH unchanged. By this replacement, bacterial cells would not continue to produce acid for the duration of the probe incubation. After incubating in the dark for 1 h to allow the probe to enter the biofilm, pH measurements of the biofilms were taken. The biofilm structure in each field was evenly divided into eight sections from the bottom to the top for Z-scanning, yielding eight slices in total. Fresh culture medium was then introduced, allowing the biofilm to continue growing for another 2.5 h, after which the aforementioned procedure of replacing medium with PBS solutions at pH 4.0 was repeated to measure the pH of the biofilm after the additional growth period in the selected fields of view. When comparing the pH of liquid regions and the surrounding microcolonies in Fig. 4d, only the surface layer was compared due to the different heights of the individual microcolonies. All experiments were performed in triplicate. All red and blue channel images were exported to ImageJ as TIFF files; the blue channel images were divided by the red channel images. The resulting ratios (R/B) were plotted against the respective pH values.

### Image processing and analysis

Images for bacterial tracking were captured using a Photometrics Prime 95B camera on an Olympus IX83 microscope (Olympus, Tokyo, Japan) with a 100× oil objective. The image size is 1,200 pixels × 1,200 pixels (132 μm × 132 μm). To quantify the bacterial adhesion ratio, 300 µL of a diluted bacterial culture (OD_600_ ~ 0.1) was injected into the flow cell chamber, allowing 20 min for bacterial adhesion. Non-adherent cells were subsequently flushed away, and the number of bacteria remaining on the surface was manually counted following the wash step. To quantify the bacterial adhesion ratio, the theoretical maximum number of cells in a microscope field of view was calculated as a normalization factor. This calculation was based on the following parameters: the bacterial concentration at OD₆₀₀ ~ 0.1 was 2.39 × 10^7^ CFU/mL; the flow cell chamber volume was 60 μL; the area of the chamber bottom was 64.6 mm^2^ (17 mm × 3.8 mm); and the area of the microscope field of view was 1.742 × 10^−2^ mm^2^ (132 μm × 132 μm). Assuming a homogeneous cell distribution, the theoretical number of cells in the field of view was determined by the formula:

Total cell numbers in chamber × (Area of one field of view/Total chamber area) ≈ 387. The adhesion ratio was therefore determined as the number of adherent bacteria within the field of view divided by 387.

Microcolonies formed at 6 h were quantified by a custom MATLAB ellipse-fitting script. The filling ratio was calculated as the ratio of the microcolony area to the area of the smallest enclosing fitted ellipse. Bright-field images of free-floating bacterial cell chains grown for 15 h were captured. The entire growth of individual bacterial chains from surface attachment to growth for 12 h was tracked. Using ImageJ, the area of each microcolony after 12 h was measured and normalized by dividing it by the initial cell number of the single-cell chain. Similarly, the height of biofilms after 12 h was obtained through Z-scan imaging and normalized by the initial cell number of the corresponding single-cell chain. The captured images were first processed using a customized MATLAB program to identify and measure the length of individual bacterial chains in the field of view. To minimize the effect of chain size on the measurements, we chose chains that have a length of 3.00 ± 0.50 µm for further analysis. Those selected chains were then skeletonized using ImageJ, and then the bending persistence length *Lp* was calculated using *Persistence* software developed by Graham et al. (55): https://delacruzlab.yale.edu/persistence-software. As previously described, MSD was calculated as ⟨Δ***r***^2^(τ)⟩ = ⟨[***r***(*t* + τ) - ***r***(*t*)]^2^⟩ = *c*τ^α^, where ***r***(*t*) represents the position vector of the cell at time *t*, and τ is the time interval; *c* is a coefficient, and α is the exponent. α > 1 indicates a superdiffusive motion, α = 1 corresponds to random Brownian diffusion, and α < 1 indicates a subdiffusive motion. We quantified the 2D-3D transition time by monitoring predefined regions of interest (ROIs) in time-lapse images. Three standardized ROI sizes were selected, corresponding to areas of approximately 10, 20, and 30 cells (based on the measured average cell area). For each ROI, we recorded the time when the focal plane of the cell chains transitioned from a dark state to a bright state (corresponding to the positional change of originally focused cells due to colony growth, i.e., 2D monolayer to 3D multilayer).

## Data Availability

All data supporting the conclusions of this study are available from the corresponding author upon request.

## SUPPLEMENTAL MATERIAL

Supplemental material aem.00009-26-s0001.pdf Fig. S1 to Fig S11, Table S1, and legends for Movies S1 to S7.

Movie S1 aem.00009-26-s0002.avi Examples showing the morphology of freely floating cell chains in BHI.

Movie S2 aem.00009-26-s0003.avi Examples showing the morphology of freely floating cell chains in BHI + 1% sucrose.

Movie S3 aem.00009-26-s0004.avi An example showing that starting from the neighboring cell of early attached cells, more and more cells in the chain changed from bright appearance to black appearance in turn, indicating that they also became attached to the surface.

Movie S4 aem.00009-26-s0005.avi An example illustrating the dynamics of fluorescent beads during the formation of liquid regions through the buckling of contacting neighboring microcolonies (bright-field with fluorescent beads overlay, corresponding to Fig. S8).

Movie S5 aem.00009-26-s0006.avi Z-scan movie of final time point (15 hours) confirming the Z-axis positions of representative fluorescent beads (corresponding to the final panel of Fig. S8 and Movie S4).

Movie S6 aem.00009-26-s0007.avi An example showing the representative three-dimensional reconstruction of *S. mutans* biofilm EPS and bacterial cells.

Movie S7 aem.00009-26-s0008.avi Bacterial cells in the liquid region display Brownian-like diffusive type of motion.

ASM does not own the copyrights to Supplemental Material that may be linked to, or accessed through, an article. The authors have granted ASM a non-exclusive, world-wide license to publish the Supplemental Material files. Please contact the corresponding author directly for reuse.
