# Supplementary material for: Role of sucrose-dependent exopolysaccharides in the biofilm development of Streptococcus mutans revealed at the microscale level
Source: Appl Environ Microbiol. 2026 Mar 3;92(4):e00009-26. doi: 10.1128/aem.00009-26 (PMC13101519; doi:10.1128/aem.00009-26)
Supplement: Supplemental material — Fig. S1 to Fig S11, Table S1, and legends for Movies S1 to S7. [file aem.00009-26-s0001.pdf]

Supplementary Materials

The role of sucrose-dependent exopolysaccharides in the biofilm development of *Streptococcus mutans* revealed at the microscale level

Ailin Huang[1,2], Xiaodan Li[1], Shangping Lu[1,2], Jingchao Zhang[3], Yujia Zheng[1], Miaoxiao Wang[1*], Kun Zhao[1,4*]

[1]Institute of Fundamental and Frontier Sciences, University of Electronic Science and Technology of China, Chengdu, Sichuan 611731, China; [2]School of Life Science and Technology, University of Electronic Science and Technology of China, Chengdu, Sichuan 611731, China; [3]College of Ecology and Environment, Chengdu University of Technology, Chengdu, Sichuan 610059, China; [4]The Sichuan Provincial Key Laboratory for Human Disease Gene Study and The Institute of Laboratory Medicine, Sichuan Provincial People's Hospital, University of Electronic Science and Technology of China, Chengdu, Sichuan 611731, China

*Corresponding author. Email: miaoxiao.wang@uestc.edu.cn (MW), kzhao@uestc.edu.cn (KZ)

The PDF file includes:

Fig S1 to Fig S11

Table S1

Legends for movie S1 to movie S7

Other Supplementary Material for this manuscript includes the following:

Movie S1 to movie S7

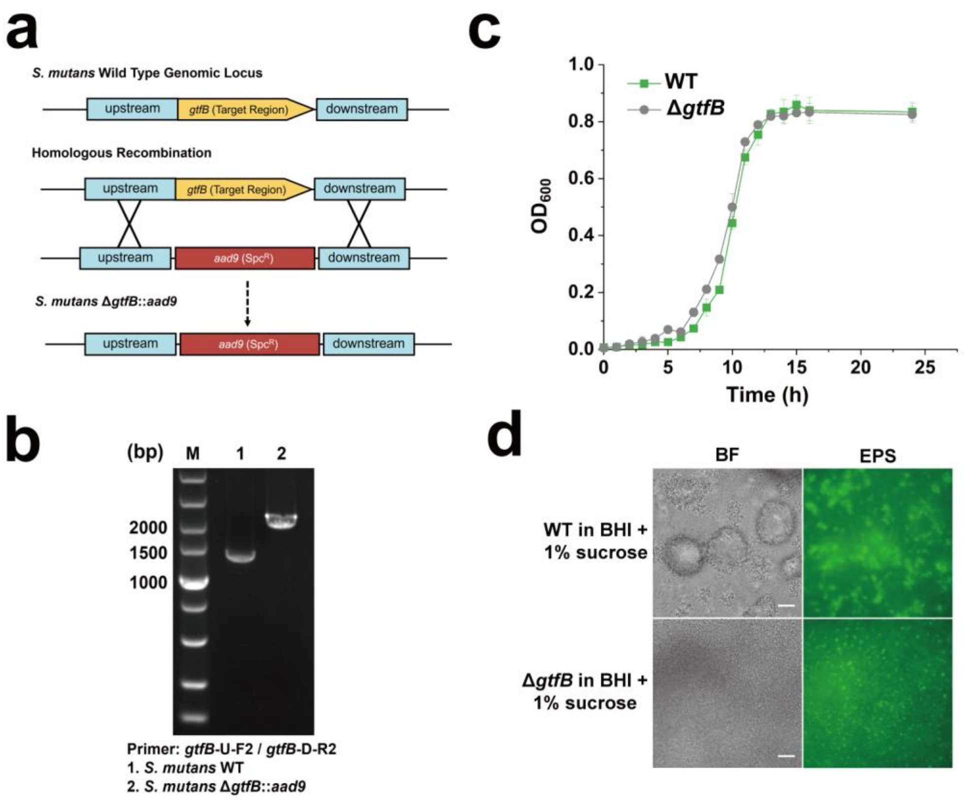

Fig S1. Construction and characterization of the *S. mutans* Δ*gtfB* mutant. (a) Schematic diagram of the strategy for constructing the *gtfB* mutant. The *gtfB* target region (525 bp) was replaced by *aad9* gene via homologous recombination using the pFW5 plasmid. (b) PCR validation of the Δ*gtfB* mutant. Representative agarose gel electrophoresis of PCR products amplified with primers gtfB-U-F2 / gtfB-D-R2. (c) Growth curves of WT and Δ*gtfB* in BHI. Data are mean ± s.d. (*n* = three independent experiments). (d) Representative images showing the biofilm phenotype / EPS stain of WT and Δ*gtfB* in BHI + 1% sucrose after 24 hours of growth in dishes. Scale bars, 10 μm.

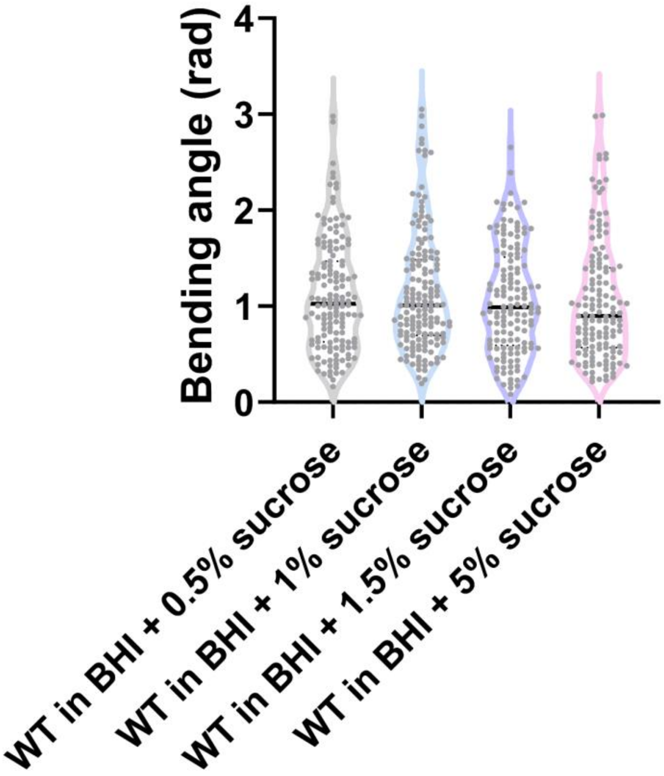

Fig S2. Quantification of the bending angle of attached WT cell chains at different sucrose concentrations. Lower angles indicate greater bending. The number of analyzed cell chains: *n* = 32 (6, 8, and 18 cell chains respectively from each experiment) for 0.5% sucrose, *n* = 27 (6, 9, and 12 cell chains respectively from each experiment) for 1% sucrose, *n* = 34 (13, 12, and 9 cell chains respectively from each experiment) for 1.5% sucrose, *n* = 28 (8, 12, and 8 cell chains respectively from each experiment) for 5% sucrose.

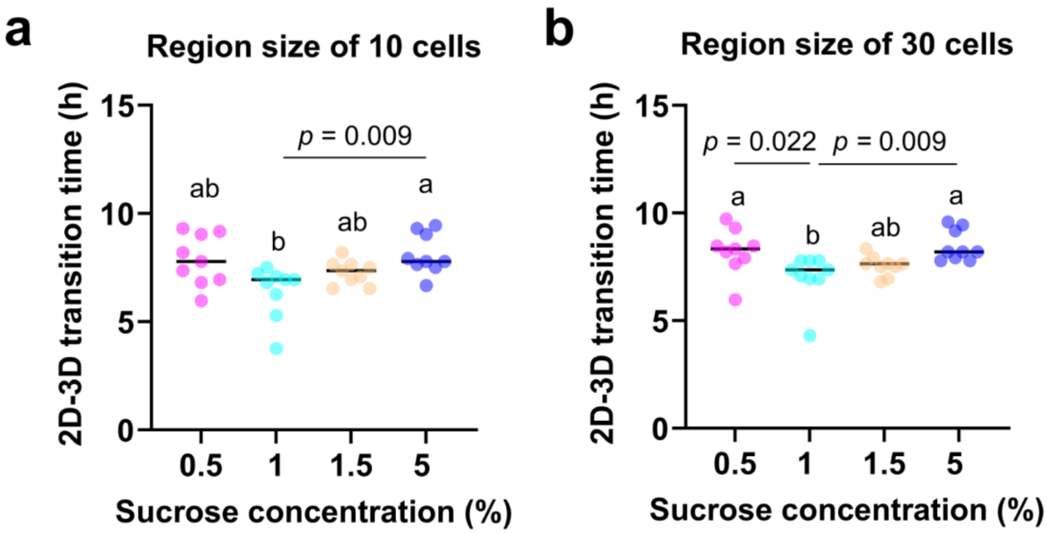

Fig S3. Measured transition times of bacteria at varying sucrose concentrations. (a) Transition times obtained by choosing the center region inside the microcolony with an area corresponding to the total area of 10 cells. (b) Transition times obtained by choosing the center region inside the microcolony with an area corresponding to the total area of 30 cells. Data are from three independent experiments, $n = 9$ cell chains (three cell chains from each experiment). Different lowercase letters (a, b, c) indicate statistically significant differences between groups ($p < 0.05$).

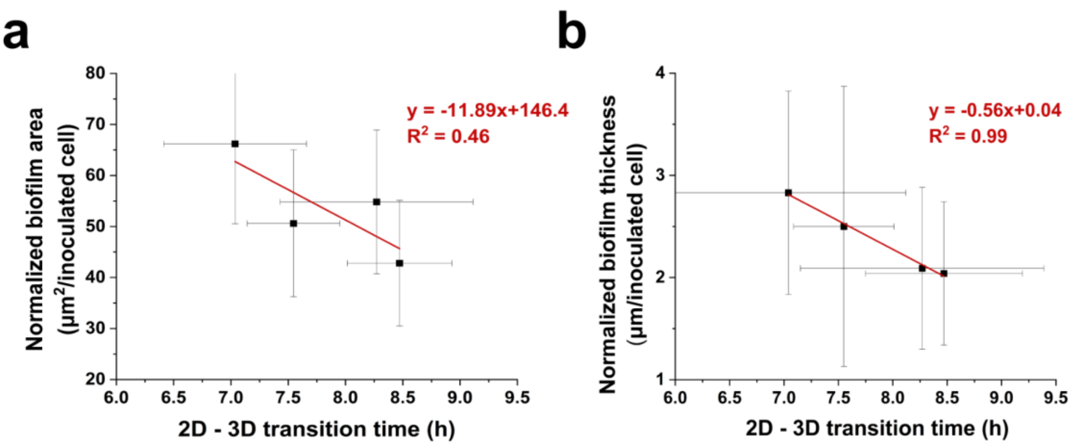

Fig S4. (a) Area and (b) thickness of a more developed biofilm v.s. its 2D-3D

transition time. The data are the same as in Fig 2. Lines are linear fitted results. Data are mean + s.d. $n = 9$ for 2D-3D transition time (three cell chains from each experiment), $n = 14$ for area and thickness (8, 3, and 3 cell chains respectively from each experiment).

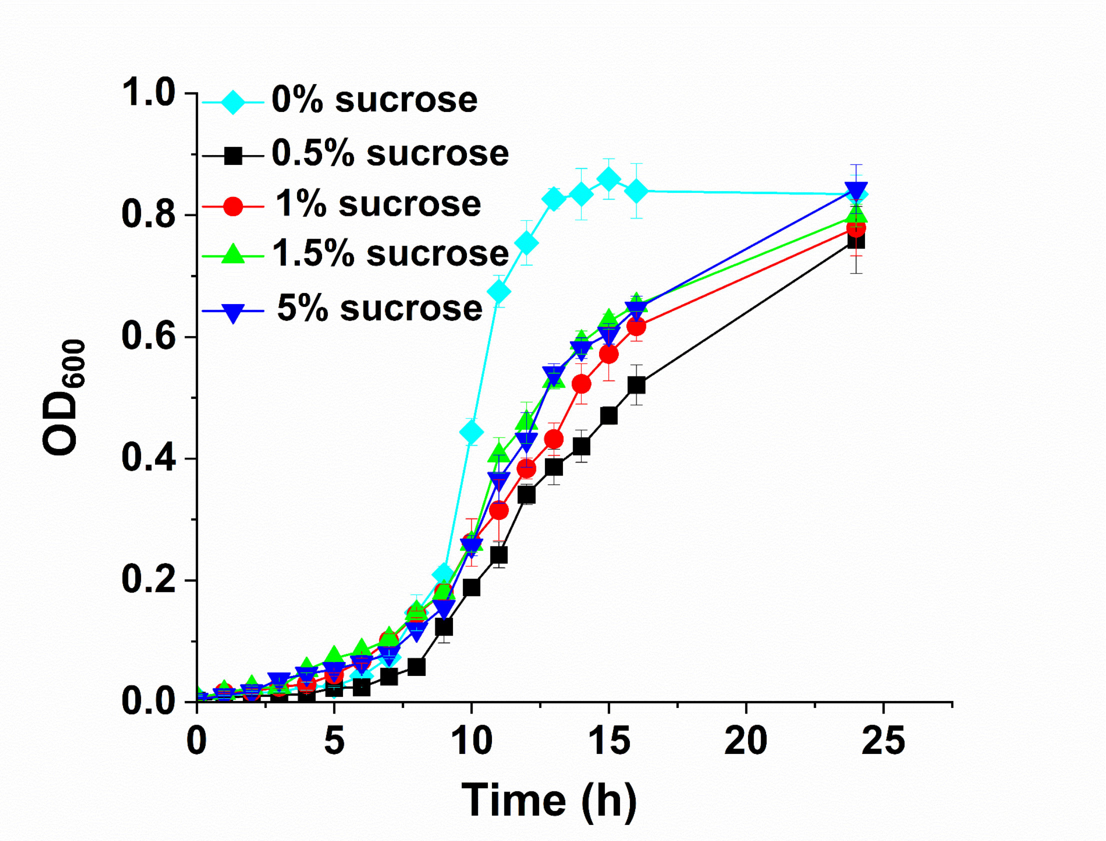

Fig S5. Growth curves of WT at different sucrose concentrations. Data are mean ± s.d. (n = three independent experiments). The sucrose supplementation delayed the entry into the logarithmic phase compared to a sucrose-free medium. This phenomenon is consistent with a previous study (1) indicating that metabolic resources are diverted towards EPS production.

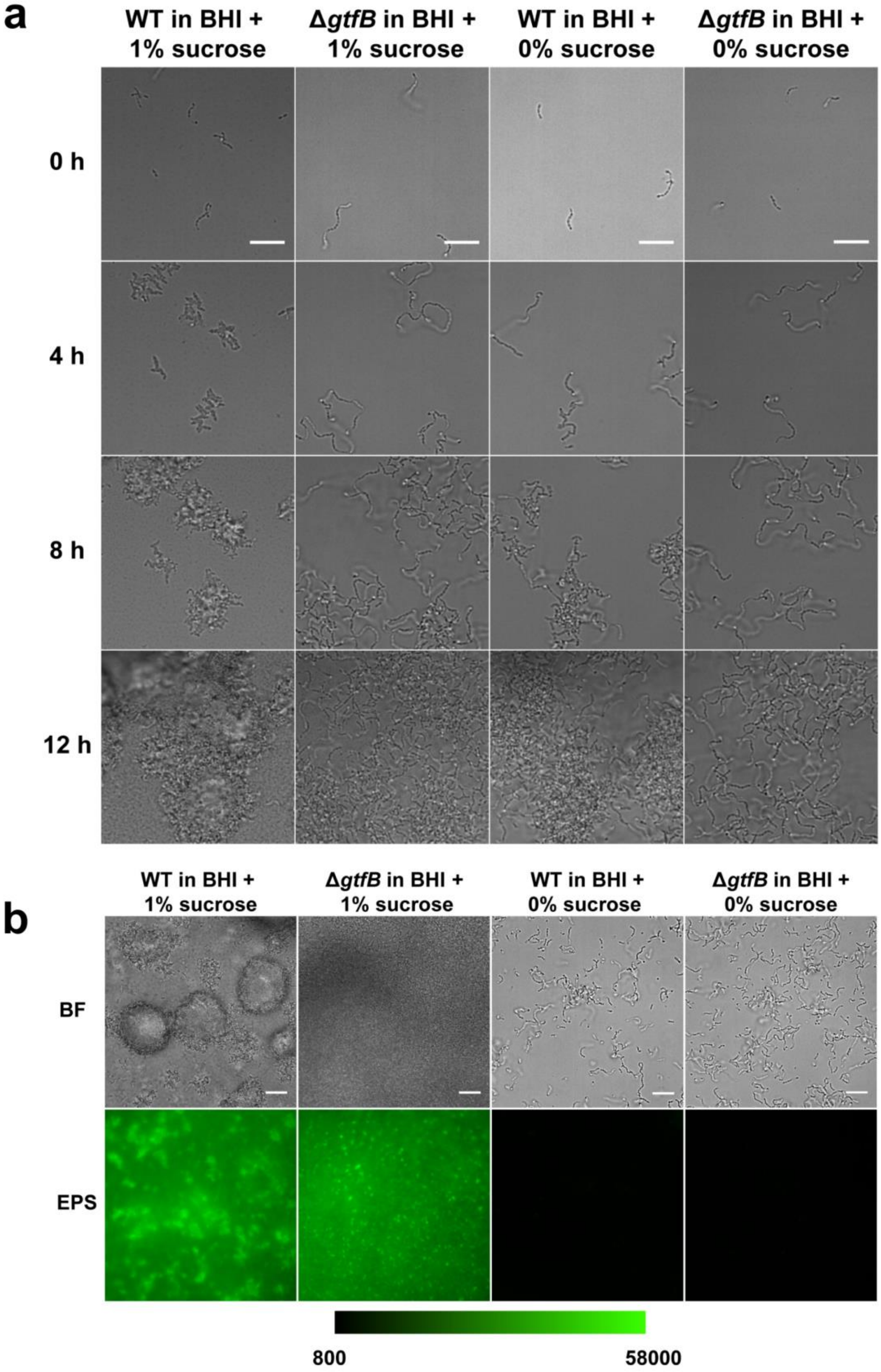

Fig S6. EPS plays a crucial role in forming the three-dimensional structure of biofilms. (a) The biofilm development process of WT and Δ*gtfB* in BHI + 1% sucrose and BHI, over a 12-hour period in a flow cell. (b) The EPS production of WT and Δ*gtfB*

in BHI + 1% sucrose and BHI after 24 hours of growth in dishes. For direct comparison, the range of grey values of the fluorescence images were manually adjusted to 800-58000. Scale bars, 10 μm.

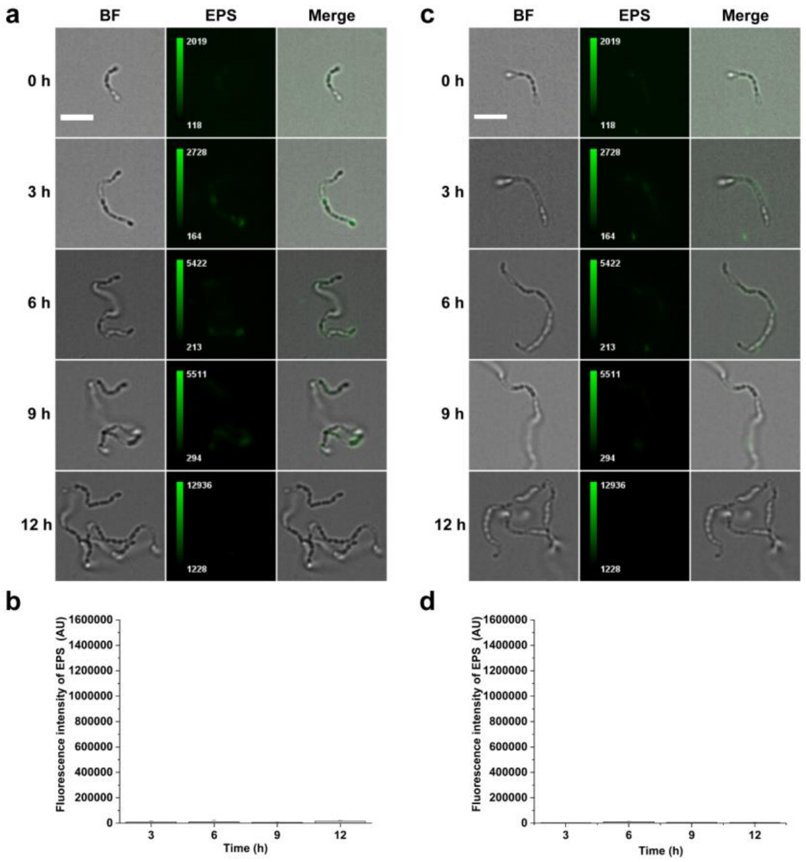

Fig S7. Dynamic measurements of glucan production of WT and Δ*gtfB* in BHI. (a) Time-lapse micrographs illustrating the glucan production of *S. mutans* WT cells in BHI. Color bars represent the fluorescence intensity range. To facilitate direct visual comparison, the range of grey values of the fluorescence images at each time point was manually adjusted to be consistent with Fig 3a. Scale bar, 5 μm. (b) The total fluorescence intensity of glucans produced by WT cells in the observed fields was measured at different time points in BHI. Data are mean ± s.d. (three fields of view from three independent experiments were analyzed). (c) Time-lapse micrographs

illustrating the glucan production of *S. mutans* Δ*gtfB* cells in BHI. Color bars represent the fluorescence intensity range. To facilitate direct visual comparison, the range of grey values of the fluorescence images at each time point was manually adjusted to be consistent with Fig 3a. Scale bar, 5 μm. (d) The total fluorescence intensity of glucans produced by Δ*gtfB* cells in the observed fields was measured at different time points in BHI. Data are mean ± s.d. (three fields of view from three independent experiments were analyzed).

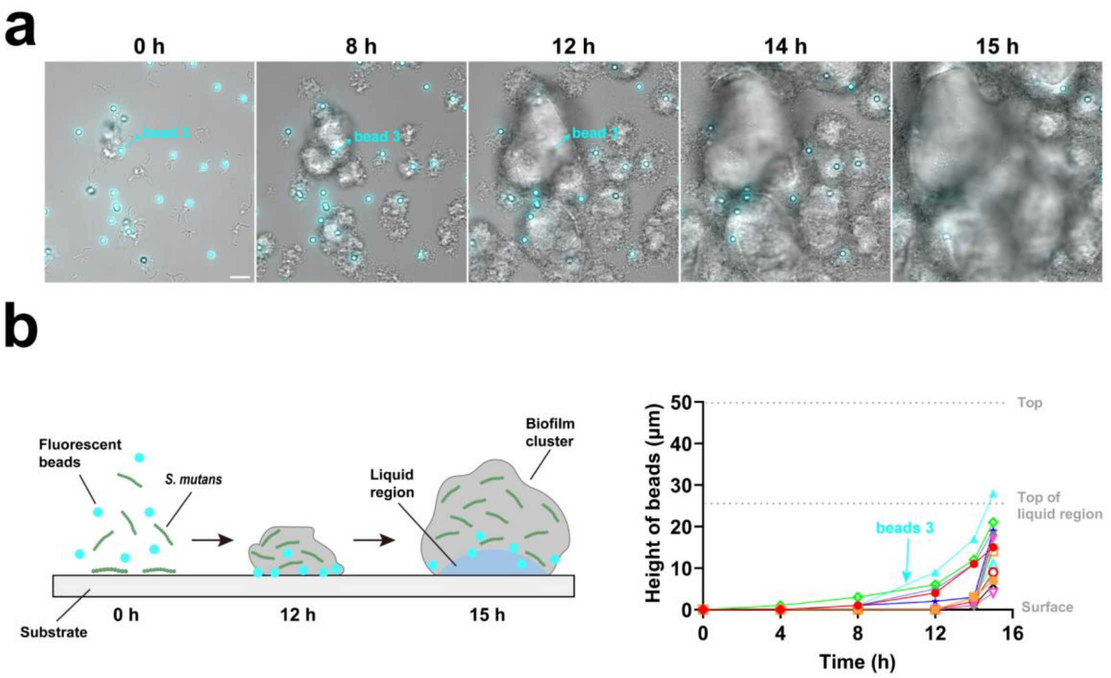

Fig S8. Fluorescent beads tracking has revealed the formation of liquid regions during biofilm development. (a) Representative time-lapse images (bright-field with fluorescent beads overlay). Fluorescent beads are shown in cyan; arrows label an example bead (bead 3) that moves upward as the biofilm develops. Scale bar, 10 μm. (b) Schematic illustration (left) showing the process of fluorescent bead incorporation. On the right, Z-height over time of the tracked 14 fluorescent beads in the same field.

Each colored line represents an individual bead. Bead 3 (cyan) corresponds to the example highlighted in (a).

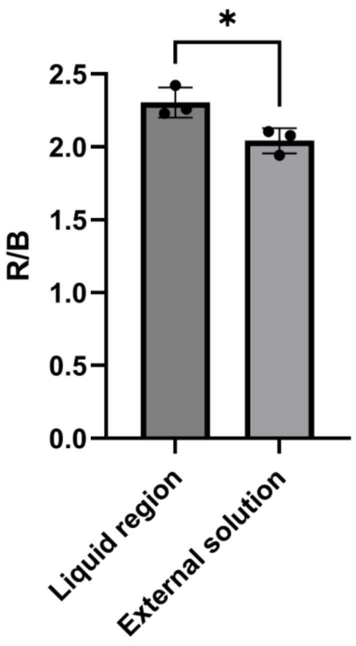

Fig S9. pH of a liquid region enclosed by colonies and the external solution measured at 12 hours after inoculation. Data are mean ± s.d. (*n* = three independent experiments). Statistical significances were measured using Student's t-test. *, $p < 0.05$.

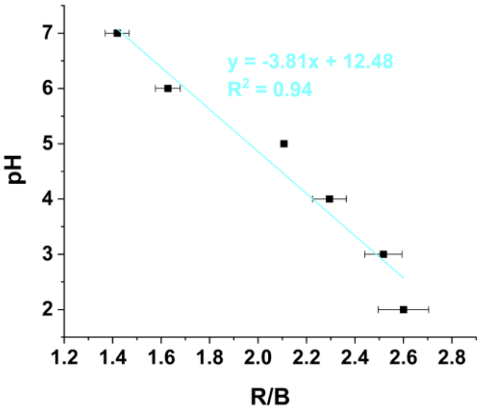

Fig S10. Titration curve used to convert R/B to pH values. Data are mean ± s.d. (*n* = three independent experiments).

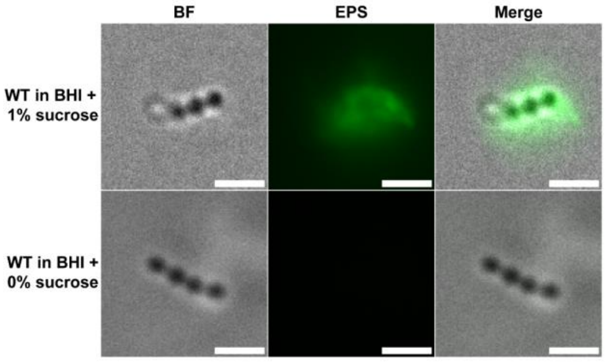

Fig S11. Fluorescent images showing the stained results of freely floating cell chains after 15 hours cultivation with and without sucrose. EPS stained with 1μM Alexa Fluor 488-labeled dextran conjugate show green. Scale bars, 5 μm.

Table S1. Primers used in this study.

Primers	Sequences (5’→3’)	Target
pF-L1-F	GTCGACGTCCGGATCCTCG	plasmid linearization
pF-L1-R	GGCCGTACGCTAGCAATTGT	plasmid linearization
gtfB-U-F	acaattgctagcgtacggccTTCATTATCGTAAAGCACTGCTC	Upstream homologous arm for *gtfB*
gtfB-U-R	tcgaggatccggacgtcgacGCTGATGGCAAAATTTTACATTT	Upstream homologous arm for *gtfB*
pF-L2-F	ATGCATCCGGAGTTCCCGGG	plasmid linearization
pF-L2-R	ATGCATGCTGCAGGGCCCATG	plasmid linearization
gtfB-D-F	atgggccctgcagcatgcatGATGTCGCTTCGGTTGTTAC	downstream homologous arm for *gtfB*
gtfB-D-R	cccgggaactccggatgcatACGCAAGTAACTAACTCTGTCG	downstream homologous arm for *gtfB*
gtfB-U-F2	TTCATTATCGTAAAGCACTGCTC	verification of *gtfB* knockout
gtfB-D-R2	ACGCAAGTAACTAACTCTGTCG	verification of *gtfB* knockout

Appendix Movie legends

Movie S1. Examples showing the morphology of freely floating cell chains in BHI. The movie was taken at a frame interval of 5 s and was played back at 10 fps. Scale bar, 5 μm.

Movie S2. Examples showing the morphology of freely floating cell chains in BHI + 1% sucrose. The movie was taken at a frame interval of 5 s and was played back at 10 fps. Scale bar, 5 μm.

Movie S3. An example showing that starting from the neighboring cell of early-attached cells, more and more cells in the chain changed from bright appearance to black appearance in turn, indicating that they also became attached to the surface. The movie was taken at a frame interval of 5 s and was played back at 100 fps.

Movie S4. An example illustrating the dynamics of fluorescent beads during the formation of liquid regions through the buckling of contacting neighboring microcolonies (bright-field with fluorescent beads overlay, corresponding to S8 Fig). Fluorescent beads are indicated with cyan markers. The movie was taken at a frame interval of 1 h and was played back at 10 fps.

Movie S5. Z-scan movie of final time point (15 hours) confirming the Z-axis

positions of representative fluorescent beads (corresponding to the final panel of S8 Fig and S4 movie). The Z-scan was acquired at 1 μm intervals over the entire biofilm thickness. The movie was played back at 10 fps.

Movie S6. An example video showing the representative three-dimensional reconstruction of *S. mutans* biofilm EPS and bacterial cells. Confocal Z-stack images of a 14.5-hour biofilm were acquired at 1 μm intervals over the entire biofilm thickness following dual staining: EPS with Alexa Fluor 488–dextran conjugate (green) and bacterial membranes with FM4-64 (red). The Z-stack was reconstructed into a 3D volume using ZEN 3.4. Playback shows rotation around the x-axis over 360°.

Movie S7. Bacterial cells in the liquid region display Brownian-like diffusive type of motion. The movie was taken at a frame interval of 0.1 s and was played back at 10 fps.

References

(1) Zhang H, Xia M, Zhang B, Zhang Y, Chen H, Deng Y, Yang Y, Lei L, Hu T. 2022. Sucrose selectively regulates Streptococcus mutans polysaccharide by GcrR. Environmental Microbiology 24:1395-1410.
